# Development of a New Route for the Immobilization of Unmodified Single-Stranded DNA on Chitosan Beads and Detection of Released Guanine after Hydrolysis

**DOI:** 10.3390/molecules28052088

**Published:** 2023-02-23

**Authors:** Ikram Chahri, Abdelhafid Karrat, Hasna Mohammadi, Aziz Amine

**Affiliations:** Laboratory of Process Engineering & Environment, Faculty of Sciences and Techniques, Hassan II University of Casablanca, P.A.146, Mohammedia 28810, Morocco

**Keywords:** DNA immobilization, guanine molecules, hydrolysis, differential pulse voltammetry, chitosan beads, electrochemical biosensors

## Abstract

In this work, chitosan beads were used as a cost-effective platform for the covalent immobilization of unmodified single-stranded DNA, using glutaraldehyde as a cross-linking agent. The immobilized DNA capture probe was hybridized in the presence of miRNA-222 as a complementary sequence. The target was evaluated based on the electrochemical response of the released guanine, using hydrochloride acid as a hydrolysis agent. Differential pulse voltammetry technique and screen-printed electrodes modified with COOH-functionalized carbon black were used to monitor the released guanine response before and after hybridization. The functionalized carbon black provided an important signal amplification of guanine compared to the other studied nanomaterials. Under optimal conditions (6 M HCl at 65 °C for 90 min), an electrochemical-based label-free genosensor assay exhibited a linear range between 1 nM and 1 µM of miRNA-222, with a detection limit of 0.2 nM of miRNA-222. The developed sensor was successfully used to quantify miRNA-222 in a human serum sample.

## 1. Introduction

Chitosan is a biopolymer that is extracted from seafood waste, mostly from the carapace of shrimps [1,2]. Indeed, the valorization of this waste by the extraction of chitin and its deacetylation in a basic medium has led to the production of chitosan [3]. This material exhibits several natural properties such as non-toxicity, biocompatibility, biodegradability, etc. [4]. Due to its reactive group’s amine and hydroxyl groups, chitosan can be used for the preparation of different materials including nanoparticles, beads, films, nanofiber, etc., through chemical modification [5,6]. Chitosan has several applications in food [7], pharmaceutical [8], cosmetics [9], agriculture [10], and the development of sensors [11] and biosensors. In fact, it can be used for the construction of electrochemical biosensors as a film for the fixation of biomolecules, including enzymes [12], antibodies [13], and DNA [14]. The immobilization of biomolecules such as DNA can be performed by entrapment, electrostatic interactions, or with covalent bonds [15]. In an acidic medium, the chitosan amino groups (-NH_2_) are protonated to Chit-NH_3_^+^ [16], which interacts electrostatically with negatively charged groups of the desired biomolecules (the anionic phosphate groups of the DNA backbone as an example). Under mild conditions, chitosan can be used as an excellent platform for covalent DNA immobilization using arm linkers such as glutaraldehyde, which links chitosan film and DNA by forming covalent imine bonds [17].

DNA biosensors have recently attracted considerable interest, owing to their high sensitivity, selectivity, cost-effectiveness, and miniaturization capability, for the determination of target DNA sequences (miRNAs). The construction of a DNA biosensor is composed of different steps: firstly the preparation of the immobilization platform, secondly the immobilization of the probe, thirdly the recognition of the miRNA by hybridization with a probe, and finally the detection of the biological recognition [18]. The procedure for immobilizing the probe on the platform plays a very important role in the construction of a biosensor. Several platforms have been used for biosensor manufacturing, including AuNPs [19], carbon sphere-MoS_2_ [20], glass microbeads [21], and amine-function-modified polystyrene 96-well microplates [22]. The ssDNA capture probe is generally modified with thiol [-SH] [23] or amine [-NH2] [24] groups at the 5’ end to covalently link with gold nanoparticles or the carboxylic functions of carbon electrodes, respectively. However, modification of the ssDNA capture probe with specific groups used for covalent immobilization can be a painstaking and expensive process. Immobilization of an unmodified ssDNA capture probe through the amine function of its nitrogen bases may therefore be an attractive and less expensive alternative to the traditional DNA method. Recently, nanomaterials have been widely used for genosensor signal amplification [25]. This is due to their unique physicochemical characteristics such as large surface area, high catalysis, and ease of electron transfer. Many kinds of nanomaterials have been used for the development of biosensors, including platinum and silver nanoparticles [26], multiwalled carbon nanotubes [27], and carbon black [28]. Carbon black as a low-cost (1 euro/Kg) nanomaterial has gained much attention in recent years because of its excellent conductivity and porosity and large specific area [29]. Recently, the functionalization of carbon nanomaterials has been the subject of research, as these materials can offer a large number of functional groups and active sites on the surface [30]. There are various innovative techniques for the detection of DNA targets; some are based on DNA labeling with nanozyme [31], CdS nanoparticles [32], or redox indicators [33], and others are unlabeled and are based on direct detection of free redox indicators [34] or guanine bases [35]. The last approach, based on guanine oxidation, is the simplest one because guanine has a high oxidation signal at the lowest ionization potential compared to other DNA constituents [36].

To the best of our knowledge, chitosan beads have not been used for the immobilization of DNA. In this study, chitosan beads were prepared for simple probe immobilization, as well as application for selective and sensitive determination of miRNA-222. MiRNAs are short non-coding RNAs that participate in the post-transcriptional regulation of gene expression in organisms. Their dysregulation is associated with a wide range of diseases such as cancers. MiRNA 222 was chosen as a model biomarker sequence because of its involvement in brain, lung, and liver cancers [37]. The amino groups of the chitosan bead platform were exploited for the covalent fixation of an unmodified DNA probe using glutaraldehyde as an arm linker. The double strands formed after the hybridization of complementary miRNA with the DNA probe sequence undergo an acid treatment to release guanine molecules, which facilitates their oxidation. A screen-printed electrode (SPE) modified with functionalized carbon black (f-CB) was used as an electrochemical platform for the oxidation of released guanine molecules by differential pulse voltammetry (DPV). Thus, a label-free detection method of miRNA is provided for analyzing microRNA-222 in (fortified) serum samples.

## 2. Results and Discussion

### 2.1. Electrochemical Characterization of Nanomaterial-Modified SPE

The electrochemical characterization of an SPE modified with different nanomaterials was evaluated with CV in a mixture of the [Fe(CN)_6_] ^3−/4−^ redox couple.

**Figure 1:**
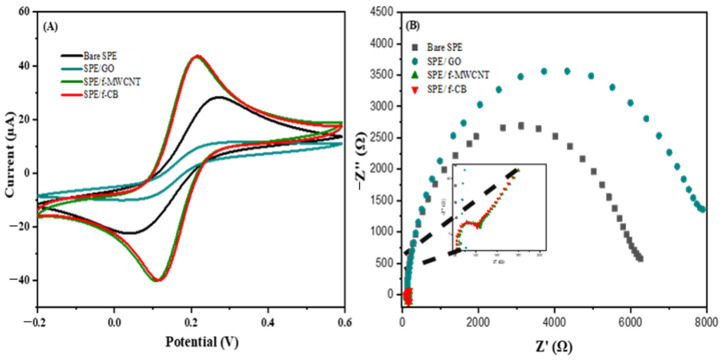
Cyclic voltammetry (**A**) and electrochemical impedance spectroscopy (**B**) of bare SPE, SPE/GO, SPE/f-MWCNT, and SPE/f-CB in 5 mM of [Fe(CN)_6_] ^3−/4−^ and 0.1 M KCl solution.

As depicted in Figure 1, various ΔE_p_ values were calculated based on the cyclic voltammetry values for a bare SPE, SPE/GO, SPE/f-MWCNT, and SPE/f-CB, which are equal to 192 mV, 168 mV, 96 mV, and 96 mV, respectively. The values of the redox peaks related to the bare SPE, SPE/GO, SPE/f-MWCNT, and SPE/f-CB were (24.51 µA; −20.16 µA), (8.91; −8.96 µA), (41.65 µA; −40.23 µA), and (41.48 µA; −40.48 µA), respectively. The modification of the SPE with different types of nanomaterials leads to the decrease of the ΔEp value, which indicates that they caused a reversible behavior. The modification of the SPE using GO showed a diminution of the current intensity due to the high resistance of charge transfer, compared to that obtained with other nanomaterials (Figure 1B), which confirms the poor conductivity of the oxygenated graphene [38]. The increase of the anodic and cathodic peak current values and the decrease of Rct after modification of the SPE by f-CB and f-MWCNT indicates excellent electron transfer on the electrode surface (Figure 1A,B). Due to the cost-effectiveness of functionalized carbon black, it was chosen for the following experiments.

### 2.2. Effect of Buffer Concentration and Modification of SPE with f-CB in the Electrochemical Oxidation of Guanine

**Figure 2:**
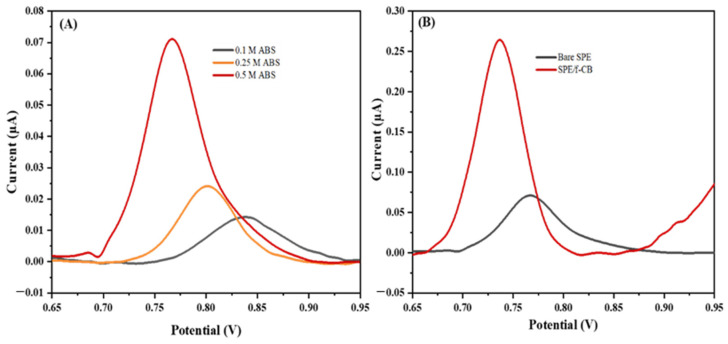
(**A**) DPV voltammograms of guanine (1 µM) in bare SPE using various concentrations of ABS between 0.1 M and 0.5 M (pH 5; 0.1 M KCl). (**B**) DPV voltammograms of bare SPE (black curve), and f-CB/SPE (red curve) using 1 μM of Guanine in 0.5 M ABS (pH 5.0).

We investigated the effect of various concentrations of ABS on guanine oxidation on a bare SPE using DPV. As indicated in Figure 2A, the current intensities of guanine (1 µM) increased with the increase of ABS concentration from 0.1 M to 0.5 M, due to the increase in ionic strength. 

The electrocatalytic activity of the treated CB in response to the oxidation of guanine was tested by the oxidation of guanine molecules (1 µM) on an SPE/f-CB and a bare SPE in ABS using the DPV technique.

As shown in Figure 2B, the current intensity at the bare SPE was 0.073 µA at an oxidative potential of 0.768 V. However, for the SPE/f-CB electrode, the peak current intensity showed a significant increase (0.275 µA), with a negative shift of the anodic peak to +0.736 V. Indeed, the negative electric charges of the carbon black nanomaterial may attract more of the positively charged guanine molecules in ABS (pH 5) [39,40], Moreover, the higher surface area and the high number of active sites improved electron transfer, increasing the peak intensity [41].

### 2.3. DNA Hydrolysis

To confirm the importance of the hydrolysis of DNA for the detection of the released guanine, the electrochemical response of 10 μM of ssDNA was investigated with and without hydrolysis using an SPE/f-CB.

**Figure 3:**
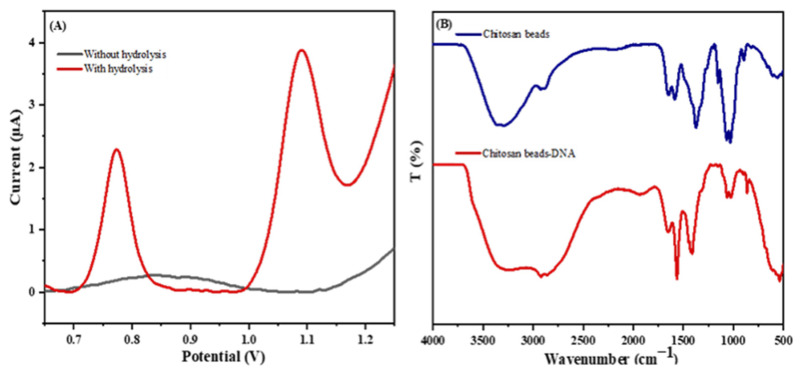
(**A**) DPV voltammograms for the electrochemical response of 10 μM of ssDNA with and without hydrolysis using SPE/f-CB in 0.5 M ABS (pH 5.0). (**B**) FT–IR spectra of chitosan beads and chitosan bead DNA.

Figure 3A shows that the peak current intensity of 10 µM without hydrolysis exhibited a low response of about 0.261 µA. Moreover, using hydrolysis, a high increase in peak intensity of guanine was obtained (2.320 µA), which could be explained by the easier oxidation of guanine molecules liberated after hydrolysis in direct contact with the SPE/f-CB. This proved the importance of DNA hydrolysis for guanine dissociation. Furthermore, in an acidic environment, the guanine molecules are positively charged and the electrode is negatively charged (CB-COO-), causing an electrostatic attraction between them. In the absence of hydrolysis, the guanine molecules are engaged in the negatively charged DNA strand backbone due to the phosphate groups, which create an electrostatic repulsion with the SPE/CB-COO-. As a consequence, the amplification of the signal observed due to the hydrolysis allows the detection of miRNA at a concentration lower than 1 nM.

DNA immobilization on the chitosan beads was confirmed by infrared spectroscopy (FT-IR). The FT-IR spectra of chitosan beads and chitosan bead DNA are shown in Figure 3B. In the case of chitosan beads, the absorption peaks of the NH and -OH stretching are at 3361 and 3291 cm^−1^; the absorption bands of C-H symmetric and asymmetric stretching are at 2921 and 2877 cm^−1^, respectively; the NH_2_CO group at 1645 cm^−1^; the absorption peak at 1589 cm^−1^, corresponding to NH_2_ bending (primary amine); and the C-O stretching at approximately 1066 cm^−1^. The FTIR spectrum of the chitosan bead DNA showed the peaks broadening at 3100–3400 cm^−1^ (NH stretching vibration present in the nitrogenous DNA bases present in DNA) and 1556 cm^−1^ for the corresponding imine (C=N) because of the DNA binding on the chitosan. The FTIR results clearly confirmed the immobilization of the chitosan beads.

### 2.4. Optimization of the Concentration of DNA Capture Probe

The miRNA-222 detection is performed by optimization of the DNA capture probe concentration. The capture probe immobilized on chitosan beads, before and after hybridization, was examined after hydrolysis using DPV in ABS (0.5 M, pH 5) in the presence of 0.1 M of KCl and the SPE/f-CB electrodes to evaluate released guanine. To obtain the best hybridization condition, various concentrations of the immobilized capture probe were tested before and after hybridization with 2 µM of a miRNA-222 target.

**Figure 4:**
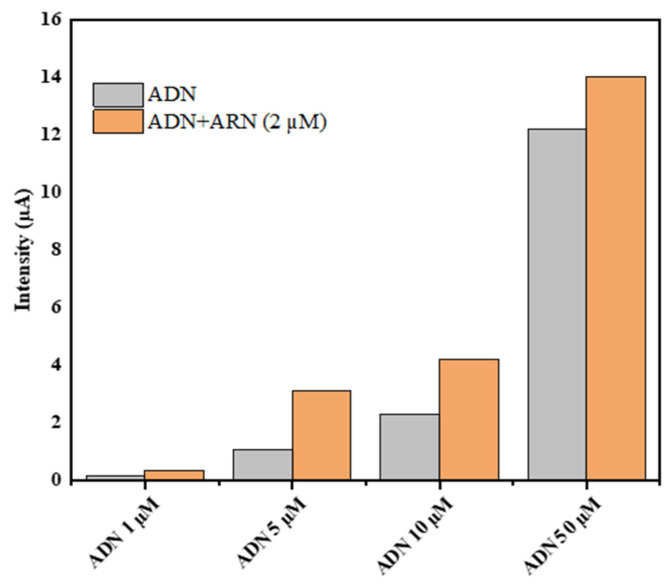
Histograms of current intensity for various probe concentrations ranging from 1 µM to 50 µM before and after hybridization using 2 µM of target DNA.

As shown in Figure 4, the differences in peak intensity (∆I) before and after hybridization for the three tested probe concentrations of 5 µM, 10 µM, and 50 µM were almost equal, but the difference obtained using 1 µM was almost negligible compared to the other concentrations. The decreased response could be explained by the decrease in the DNA concentration of the probe, resulting in a decrease in the number of hybridization sites. Ultimately, 5 µM was used as the appropriate concentration of the capture probe for the rest of this study because it was the smallest concentration that produced good hybridization results. 

### 2.5. Electrochemical Detection of miRNA-222

The analytical efficiency of the suggested biosensor was investigated as follows: after hybridization of the immobilized probe on chitosan beads with different concentrations of miRNA-222, the beads were monitored using a 6 M HCl acid treatment at 65 °C until complete drying. Guanine was released for electrochemical detection by DPV using an SPE/f-CB and 0.5 M ABS (pH 5). After complete acidic drying, 30 µL of 0.5 M ABS (pH 5) was added to dissolve the released guanine to allow electrochemical evaluation.

**Figure 5:**
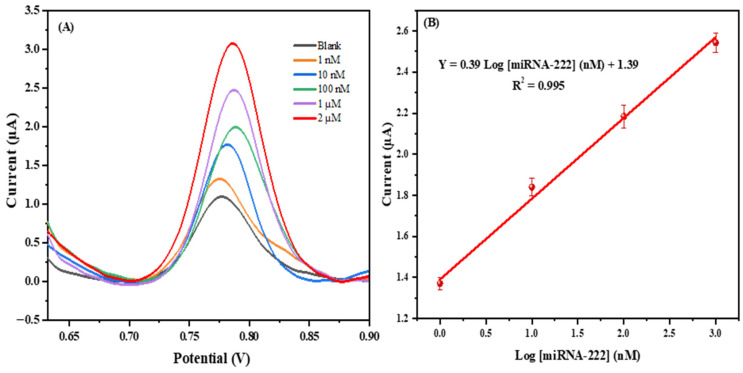
(**A**) DPV voltammograms of the developed biosensor at various concentrations of miRNA-222 (from 1 nM to 2 µM) using SPE/f-CB. (**B**) Calibration curve for miRNA-222 detection.

Figure 5 shows that the peak intensity of the guanine oxidation increased with the concentration of the target (miRNA-222). The voltammograms of the developed biosensor at various concentrations of miRNA-222 (from 1 nM to 2 µM) using an SPE/f-CB are presented in Figure 5A. The peak current values were linearly related to the logarithm of the guanine concentrations (from1 nM to 1 µM), with a correlation coefficient of about 0.995 and a regression equation of I = 0.39 Log [miRNA-222] (nM) + 1.39 (Figure 5B). The obtained limit of detection (LOD) was 0.2 nM estimated using the equation 3 sb/σ, where sb is the standard deviation of the blank measurements and σ is the slope of the calibration plot.

It is known that the covalent immobilization strategy has significant advantages, such as high bounding strength, compared to other strategies such as adsorption [42,43,44] or biotin-avidin interaction [45,46,47]. However, it presents some disadvantages, such as the necessity of modification of the probe DNA with functional groups using a tedious and expensive process. In this study, the results of the immobilization of unmodified DNA on chitosan beads using a cross-linking agent were compared to the results of previous work. As can be seen in Table 1, the sensitivity of the proposed biosensor is comparable to biosensors requiring other immobilization procedures and detection techniques. Moreover, the presence of intercalants, as well as the use of gold electrodes, ITO, and MWCNTs, make these methods more expensive than our method, based on the DPV oxidation of released guanine after DNA hydrolysis.

### 2.6. Repeatability

To examine the repeatability of the proposed biosensor, the relative standard deviation (RSD%) of three repeated measurements of the intensity of various miRNA-222 target concentrations between 1 nM and 1 μM was evaluated. The RSD value of the calibration curve slope was 5.05%, indicating the potential of the use of the designed biosensor for miRNA-222 detection.

### 2.7. Selectivity Study

The selectivity of the developed biosensor towards miRNA-222 was investigated by measuring the response of the miRNA-21 as a non-complementary sequence of probe miRNA-222.

**Figure 6:**
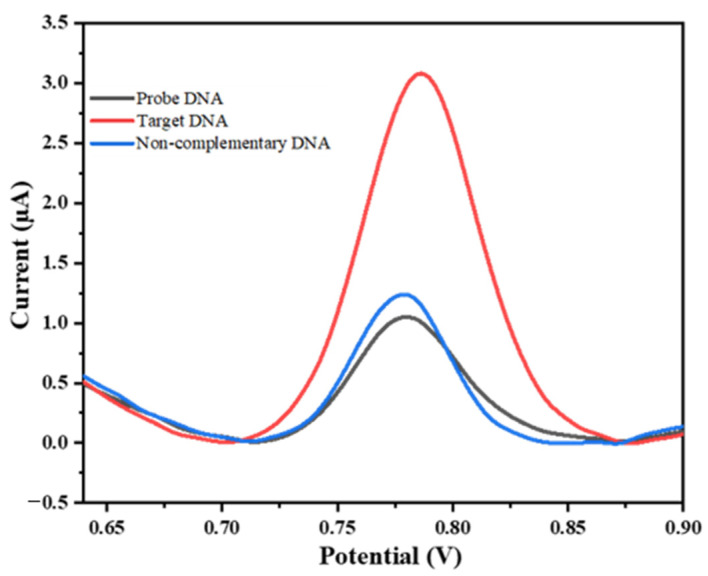
DPV responses of the developed biosensor in the presence of 2 µM of perfectly matched target miRNA-222 (red curve), compared to 2 µM of non-complementary sequence target RNA (blue curve), after hydrolysis in 0.5 M ABS (pH 5) using SPE/f-CB.

As can be seen in Figure 6, the intensity of guanine oxidation in response to perfectly matched target miRNA (3.14 ± 0.063 µA) was significantly higher than for the non-complementary target RNA (1.22 ± 0.028 µA), which was nearly equal to the value obtained with the ssDNA probe. This confirms the high selectivity of the developed method for miRNA-222 detection.

### 2.8. MiRNA-222 Analysis in Real Sample

To test the analytical performances of the proposed biosensor for the detection of miRNA-222 in real samples, various concentrations of miRNA-222 were added to diluted artificial human serum (30× in PBS). We added 60 µL of spiked serum to the chitosan beads modified with the single-strand DNA, and after the incubation period, performed the washing step before hydrolysis and electrochemical guanine analysis, as described above. It should be noted that the washing step was performed in order to avoid non-specific adsorption, which eliminates serum proteins. As depicted in Table 2, the average ∆I current obtained for three different concentrations of miRNA-222 showed good recovery, ranging between 99% and 103%, with RSD less than 3.51%. This confirmed the ability of the developed biosensor to detect miRNAs cancer biomarkers in a biological complex matrix.

## 3. Materials and Methods

### 3.1. Chemicals

Carboxylic multi-walled carbon nanotubes (f-MWCNT) (diameter: 9.5 nm) were purchased from Sigma-Aldrich (Overijse, Belgium), and graphene oxide (GO) solution (2 mg/mL) (sheet diameter: 22 µm) and chitosan were purchased from Sigma Aldrich (Saint Louis, MO, USA). Commercial-grade carbon black (CB) N220 (32 nm) was obtained from Cabot Corporation (Ravenna, Italy), and glutaraldehyde, potassium chloride (KCl), guanine, human serum, and ethanolamine were purchased from Merck (Darmstadt, Germany). Hydrochloric acid (HCl) was bought from VWR. Potassium ferrocyanide [K_4_Fe(CN)_6_], and potassium ferricyanide [K_3_Fe(CN)_6_] were purchased from Surchem products (Ipswich, UK). Sodium hydroxide and sodium acetate were purchased from Lobal Chemie (Mumbai, India). Acetic acid and all chemicals used for the preparation of the phosphate buffer saline (PBS) (137 mM NaCl, 2.7 mM KCl, 10 mM Na_2_HPO_4_, and 1.8 M KH_2_PO_4_, pH 7.4) were purchased from Solvachim (Casablanca, Morocco). All solutions were prepared with distilled water at room temperature (+20 °C). All reagents used were of analytical grade.

The oligonucleotides purified by HPLC were purchased from Eurofins Genomics (The Ulis, France) as lyophilized shapes. The sequences of oligonucleotides are as follows:

Target miRNA: 5′-AGC [U]AC A[U]C [U]GG C[U]A C[U]G GG[U] C[U]C-3′. Probe DNA (complementary sequence of target miRNA): 5′-GAG ACC CAG TAG CCA GAT GTA GCT-3′. Non-complementary sequence: 5′AGC [U][U]A [U]CA GAC [U]GA [U]G[U] [U]GA-3′.

The synthetic oligonucleotide stock solutions were prepared with distilled water and stored at 20 °C. More diluted oligonucleotide solution was prepared in PBS.

### 3.2. Apparatus

The electrochemical measurements were performed using a PalmSens 2 purchased from PalmSens BV (Houten, The Netherlands) connected to a computer and controlled with PsTrace 4.2 software.

Screen-printed electrodes were obtained from the University of Rome Tor Vergata (Italy) and were composed of a polyester film as the substrate, silver chloride as a reference electrode, and graphite as working and counter electrodes. The working electrode had a diameter of 0.3 cm and a geometric area of 0.07 cm^2^.

### 3.3. Preparation of Functionalized Carbon Black

The functionalization of CB involves, first, dispersing 1 g of CB in a mixture of concentrated sulfuric and nitric acid at a volume ratio of 1:3 (40 mL) using ultrasound irradiation. The obtained solution is then refluxed for 6 h at a temperature of 80 °C under magnetic stirring. The resultant product is then washed with distilled water until a neutral pH, collected with a centrifuge, and dried in an oven for 8 h at a temperature of 60 °C [21].

### 3.4. Preparation of Nanomaterial Dispersions

A concentration of 1 mg/mL of GO was dispersed by sonication for 1 h in distilled water. The carboxylic multi-walled carbon nanotube (f-MWCNT) dispersion was obtained by adding 10 mg of f-MWCNT to 10 mL of DMF and sonicating for 1 h. For the preparation of the f-CB dispersion, 10 mg CB-COOH was introduced into 10 mL of distilled water and sonicated for 1 h to obtain a homogeneous f-CB dispersion.

### 3.5. SPE Modification by Nanomaterials

SPEs were modified with various nanomaterial dispersions using the drop-casting technique. A total of 12 μL of the GO, f-MWCNT or f-CB dispersion was dropped (2 × 6 µL) onto the surface of the working electrode and dried at 45 °C for 10 min.

### 3.6. Electrochemical Measurements

The efficiency of the electrode surface was checked with cyclic voltammetry and electrochemical impedance spectroscopy using 5 mM [Fe (CN)_6_] ^3−/4−^ and 0.1 M of KCl. The CV measure was realized at a potential range of −0.2 V to +0.6 V, an 8 mV step potential, at a scan rate of 0.01 V/s. The EIS measure was performed at an applied potential of 0.16 V with a potential amplitude of 10 mV and a frequency range of 5 Hz to 20,000 KHz.

Moreover, DPV was used for the guanine detection in acetate buffer saline (ABS, 30 µL, 0.5 M, pH 5, 0.1 M KCl) using the following parameters: 30 mV amplitude potential, 8 mV step potential, 0.1 s pulse time and a scan rate of 0.01 V/s. Each measurement was performed with a single-use electrode.

The characterization with Fourier transform infrared (FTIR) spectroscopy was recorded with an IRAffinity-1S spectrophotometer (Shimadzu) in the range of 2000–500 cm^−1^ in the attenuated total reflectance mode.

### 3.7. Preparation of Chitosan Beads

**Scheme 1:**
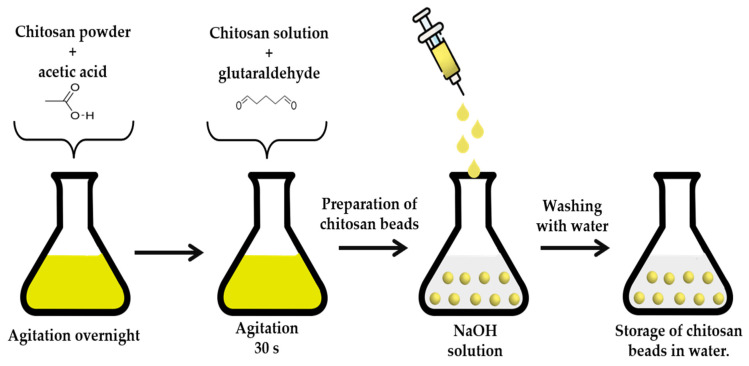
Method for the preparation of chitosan beads.

The chitosan beads were prepared as follows: 0.75 g of chitosan powder was solubilized in 50 mL of acetic acid (0.2 M), and 5 g of the prepared solution was mixed with 45 µL of glutaraldehyde (25% (*v*/*v*) for 30 s and then added dropwise to 1.5 M of NaOH (50 mL) solution using a syringe [50]. After the contact of the drops with the NaOH, the chitosan beads were formed (Scheme 1). The beads were then washed three times with distilled water to remove unreacted glutaraldehyde and NaOH.

### 3.8. Single-Stranded DNA Immobilization

Glutaraldehyde solution (2.5%, 30 µL) and an appropriate concentration of unmodified DNA probe (30 µL) were added to the surface of chitosan beads (two chitosan beads prepared as described above). Glutaraldehyde was used as a cross-linking agent for the immobilization of the capture DNA via imine group (C=N) formation at room temperature. The beads were then washed three times with PBS. Non-specific sites were then blocked for 15 min by adding 1 mM ethanolamine (60 µL). Finally, the platform was rinsed with PBS three times before the next use.

### 3.9. Hybridization of miRNA

To detect miRNA-222, the chitosan beads modified with the single-strand DNA were incubated in 60 µL of different concentrations of the complementary sequence for 1 h at room temperature. The platform was then rinsed with PBS three times before the next use.

### 3.10. Hydrolysis of Nucleic Acid Compounds and Liberation of Guanine

**Scheme 2:**
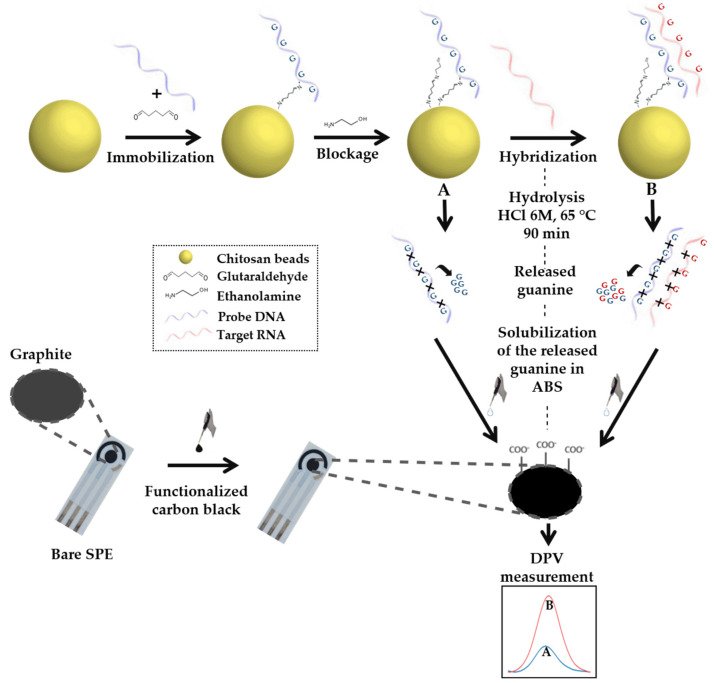
Illustration of electrochemical biosensor based on the detection of guanine released after DNA hydrolysis at SPE/f-CB.

The release of guanine from the DNA or DNA/miRNA strands was performed by hydrolysis with a 30 µL solution of 6 M of HCl at 65 °C until dryness [22] (Scheme 2).

## 4. Conclusions

The chitosan beads were successfully used as a platform for the immobilization of the unmodified DNA probe by covalent linkage. This platform allowed selective hybridization of the target biomolecule (miRNA-222). Moreover, its combination with an electrochemical sensor based on SPE modified with functionalized carbon black provided sensitive detection of miRNA-222. The DNA hydrolysis procedure improved the sensitivity of the target detection. The proposed biosensor exhibited good repeatability, indicating its accurate detection of miRNA-222. Moreover, it could be effectively applied for the determination of miRNA-222 in spiked serum samples.

## Figures and Tables

**Table 1:** Comparison of various immobilization platforms of DNA probes.

Platform	Strategies	Detection Method	Solid Substrate	Limit of Detection	Reference
PET	Adsorption of ferrocene-DNA	Current reduction of ferrocene tags by CV and DPV.	ITO	5 × 10^−9^ M	[42]
Graphene/AuNP hybrid	Adsorption of unmodified DNA	Catalytic activity of hybrids with a concomitant colorless-to-blue color change.	In solution	3.2 × 10^−9^ M	[43]
Polylysine-chitosan	Adsorption of unmodified DNA	Decrease of the signal of methylene blue by DPV	Au	4.4 × 10^−9^ M	[44]
Ar-COOH/Avidin	Avidin interaction with biotinylated DNA	Decrease of current value after hybridization using CV	GCE	8.51 × 10^−14^ M	[45]
Graphite-Avidin	Avidin interaction with biotinylated DNA	Daunomycin hydrochloride as an indicator of hybridization reaction (DPV)	SPE	1 µg/mL	[46]
MWCNT-STR	Avidin interaction with biotinylated DNA	Guanine oxidation using DPV	PGE	1.45 × 10^−7^ M	[47]
AuNWs	Covalent interaction with SH-DNA	Oxidation signal of methylene blue accumulation measured using DPV	AuNWs electrode	6.78 × 10^−9^ M	[48]
Chitosan-MWCNTs/GTD	Covalent interaction with NH_2_-DNA	Hybridization capacity monitored by EIS	GCE	8.5 × 10^−14^ M	[49]
RGO/Ar-COOH/EDC/NHS	Covalent interaction with NH_2_-DNA	Hybridization capacity monitored by EIS	SPE	1 × 10^−9^ M	[38]
Chitosan beads/GTD	Covalent interaction with unmodified DNA	DPV oxidation of released guanines	SPE	0.2 × 10^−9^ M	This work

GCE: glassy carbon electrode; GTD: Glutaraldehyde; SPE: Screen-printed electrode; STR: Streptavidin; PET: polyethylene terephthalate; ITO electrode: indium tin oxide; PGE: Pencil graphite electrode; EIS: Electrochemical impedance spectroscopy.

**Table 2:** Analytical results for miRNA-222 in a real sample using the developed genosensor (*n* = 3).

Added miRNA (nM)	Detected (nM)	Recovery (%)	RSD %
1	0.99	99.0	2.55
10	01.3	103.0	3.51
1000	986	98.6	2.02

## Data Availability

The data presented in this study are available on request from the corresponding author.
